# Risk Factors for Emergence Delirium in Elderly Orthopedic Patients After General Anesthesia

**DOI:** 10.1002/brb3.71047

**Published:** 2025-11-10

**Authors:** Yufan Lu, Lin Wang, Ying Wang, Beiyan Ruan, Guangtao Lu, Xuezheng Lin

**Affiliations:** ^1^ Department of Anesthesia Surgery Taizhou Central Hospital (Taizhou University Hospital) Zhejiang China

**Keywords:** emergence delirium, elderly patients, orthopedic surgery, risk factors

## Abstract

**Objective:**

The primary objective of this research was to investigate the occurrence rate and independent risk factors associated with emergence delirium (ED) in elderly patients undergoing orthopedic procedures with general anesthesia, providing a basis for clinical prevention and intervention.

**Methods:**

Elderly individuals (age ≥65 years) undergoing orthopedic procedures under general anesthesia with subsequent post‐anesthesia care unit (PACU) admission were included in this secondary analysis of a prospective observational study. ED was assessed at 10 and 30 min after PACU admission and immediately before discharge using the Nursing Delirium Screening Scale. Univariate logistic regression was first employed to screen potential risk factors, after which multivariate logistic regression analysis was conducted to determine independent risk factors.

**Results:**

ED was observed in 85 individuals, accounting for 35.1% of the 242 patients. Multivariate logistic regression analysis showed that the presence of urinary catheters (odds ratio [OR] 4.30, 95% confidence interval [CI] 2.08–8.91), age (OR 1.14, 95% CI 1.08–1.20), postoperative numerical rating scale score (OR 1.37, 95% CI 1.08–1.73), and fasting times for solids (OR 1.10, 95% CI 1.02–1.19) were independent risk factors for ED. The multivariate model demonstrated acceptable discrimination with an area under the curve of 0.77 and excellent calibration (Hosmer–Lemeshow test, *p* = 0.637).

**Conclusion:**

ED represents a common complication following general anesthesia in elderly orthopedic patients. Some risk factors, including the presence of urinary catheters, prolonged fasting times for solids, advanced age, and postoperative pain, can increase the incidence of ED. Perhaps we can reduce the occurrence of ED through multimodal clinical strategies.

## Graphical abstract

Emergence delirium occurred in 35.1% of elderly orthopedic patients post‐anesthesia. Independent risk factors were the presence of urinary catheters, prolonged fasting times for solids, advanced age, and postoperative pain. This study suggests that multimodal strategies targeting these modifiable factors could provide a basis for clinical prevention and intervention.

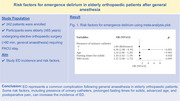

## 1 Introduction

With the intensification of the aging population, the incidence of fractures among the elderly has also increased. Delirium, as a postoperative complication in elderly patients with fractures, has become increasingly common (An et al. 2023; Wang et al. 2023). Emergence delirium (ED) is characterized by a sudden change in the level of consciousness, inattention, and cognitive dysfunction and usually occurs from the disuse of general anesthetic drugs to leaving the operating room (Marcantonio 2017; Assefa and Sahile 2019). According to the literature, ED occurs in 3.7%–45% of patients after surgery (Zhang et al. 2020; Lu et al. 2025). ED poses serious risks to patients. It may lead to the destruction of wound dressings, exacerbated pain, injury, bleeding, and unintended removal of medical devices (such as drainage tubes), which can prolong the length of hospital stay, increase the perioperative mortality rate, and complicate nursing care (Assefa and Sahile 2019; Ansaloni et al. 2010).

So far, the underlying mechanisms of delirium remain incompletely understood, and its occurrence may depend on the interaction of multiple factors (Janssen et al. 2019). Current therapeutic options for delirium remain limited, and prevention is widely regarded as the most effective management strategy. Consequently, the identification of relevant risk factors may significantly impact the prevention of ED. Nevertheless, existing data on ED remain relatively scarce, with most studies focusing on delirium development following discharge from the post‐anesthesia care unit (PACU).

Given these considerations, the present research intends to analyze the incidence and potential risk factors of ED among elderly orthopedic patients receiving general anesthesia and to discuss relevant preventive measures. These findings may provide evidence‐based support for ED risk reduction strategies in this clinical population.

## 2 Materials and Methods

### 2.1 Ethics Statements

This article presents a secondary analysis derived from a prospectively designed observational research. The Clinical Research Ethics Committee at Taizhou Central Hospital (Taizhou University Hospital) granted ethical approval (Approval No. 2023L‐11‐07), and the study was officially registered with the Chinese Clinical Trial Registry (ChiCTR2300078314). All enrolled patients or their legal guardians provided written informed consent before study participation.

### 2.2 Participants

We enrolled the data of patients who received general anesthesia in our institution between December 11, 2023 and April 30, 2024. Eligible participants were geriatric patients (≥65 years) undergoing elective orthopedic procedures under general anesthesia with an expected duration exceeding 60 min; all were planned for extubation in the operating room followed by PACU admission. Exclusion criteria comprised (1) preexisting dementia or neuropsychiatric disorders, (2) severe hearing/speech impairment precluding communication, (3) postoperative transfer to the intensive care unit, (4) history of surgery within the preceding 3 months, and (5) incompletely recorded patient data.

### 2.3 Anesthesia and Perioperative Care

Routine intraoperative monitoring in the operating room included hemodynamic parameters (electrocardiogram and blood pressure), respiratory indices (pulse oxygen saturation and end‐tidal carbon dioxide), and depth of anesthesia (bispectral index). General anesthesia was induced using etomidate (0.2–0.3 mg/kg) and/or propofol (1.5–2.5 mg/kg), sufentanil (0.3–0.6 µg/kg), and rocuronium (0.6 mg/kg). Anesthesia was performed with sevoflurane (end‐tidal concentration maintained at 0.8–1.3 MAC), with or without remifentanil (0.05–0.25 µg/kg/min) and/or dexmedetomidine (usually maintained at an infusion rate of 0.2–0.5 µg/kg/h without a loading dose). The decision to use remifentanil and/or dexmedetomidine was left to the attending anesthetist, who considered factors, such as patient comorbidities, anticipated surgical stimulus intensity and duration, and the need for perioperative hemodynamic optimization. The depth of anesthesia was maintained with a bispectral index ranging from 40 to 60. Rocuronium and sufentanil could be administered intermittently as required. At the end of the surgery, sugammadex sodium was administered to reverse residual neuromuscular block. Once extubation criteria were met, patients were extubated in the operating room and then transferred to the PACU for a minimum of 30 min of monitoring. Standardized postoperative pain evaluation was performed immediately upon arrival in the PACU utilizing the numerical rating scale (NRS), where scores ranged from 0 (no pain) to 10 (maximum conceivable pain) (Fillingim et al. 2016).

### 2.4 Study Outcomes

The study's primary endpoint focused on the prevalence of ED among patients in the PACU. ED was evaluated at three predefined intervals utilizing the Nursing Delirium Screening Scale (Nu‐DESC) (Gaudreau et al. 2005): 10 min after PACU admission, 30 min after admission, and immediately prior to PACU discharge. This scale includes five aspects: (1) illusions/hallucinations, (2) inappropriate communication, (3) inappropriate behavior, (4) disorientation, and (5) psychomotor retardation. All items are scored from 0 (absent) to 2 (severe), yielding a total score range of 0–10. A Nu‐DESC score ≥2 at any assessment time point was considered diagnostic of ED. All assessments were conducted by anesthetists or nursing staff who had undergone a unified standardized training protocol to minimize inter‐rater variability. Although formal inter‐rater reliability testing was not conducted, this training ensured consistent application of the scale.

### 2.5 Data Collection

On the basis of previous research evidence (Wang et al. 2023; Zhang et al. 2020; Li et al. 2024; Wang et al. 2023; Lai et al. 2023), we identified potential risk factors associated with ED occurrence (Table 1) and categorized them into the following three groups:

1. Preoperative factors: Demographic characteristics (body mass index, age, sex, current smoker, education, and current drinker); health status: coronary artery disease, American Society of Anesthesiologists (ASA) class; chronic obstructive pulmonary disease, hypertension, hyperlipidemia, diabetes mellitus, arrhythmia, and stroke; laboratory indicators (blood routine, renal function, blood glucose, electrolytes, and inflammatory markers such as neutrophil‐to‐lymphocyte ratio [NLR]).
2. Anesthetic and surgical factors: Fasting time, intraoperative hypothermia (defined as a core temperature <36°C measured via nasopharyngeal probe), hypotension (characterized as systolic blood pressure <90 mmHg or requirement for vasopressors in the surgery), and blood transfusion; surgical characteristics: duration of surgery, blood loss, and total fluid infusion; anesthetic agents: use of dexmedetomidine, opioids, and glucocorticoids.
3. Postoperative factors: NRS score, presence of urinary catheters.

**TABLE 1:** Demographic and perioperative data of all patients.

Variables	Total (*n* = 242)	Non‐ED (*n* = 157)	ED (*n* = 85)	*p* value
Preoperative factors				
Age, year	73 (68, 76)	71 (68, 75)	75 (71, 81)	<0.001
Body mass index, kg/m^2^	23.90 (21.48, 26.30)	23.83 (21.48, 26.30)	24.09 (21.48, 25.71)	0.824
Male, *n* (%)	97 (40.08)	60 (38.22)	37 (43.53)	0.421
Education, year	6 (0, 6)	6 (0, 6)	0 (0, 6)	0.136
Current drinker, *n* (%)	15 (6.20)	10 (6.37)	5 (5.88)	0.881
Current smoker, *n* (%)	16 (6.61)	11 (7.01)	5 (5.88)	0.737
ASA class, *n* (%)				<0.001
I	28 (11.57)	23 (14.65)	5 (5.88)	
II	190 (78.51)	127 (80.89)	63 (74.12)	
III	24 (9.92)	7 (4.46)	17 (20.00)	
Coronary artery disease, *n* (%)	7 (2.89)	2 (1.27)	5 (5.88)	0.101
COPD, *n* (%)	4 (1.65)	2 (1.27)	2 (2.35)	0.920
Hypertension, *n* (%)	138 (57.02)	81 (51.59)	57 (67.06)	0.020
Hyperlipidemia, *n* (%)	83 (34.30)	62 (39.49)	21 (24.71)	0.021
Diabetes mellitus, *n* (%)	40 (16.53)	26 (16.56)	14 (16.47)	0.986
Arrhythmia, *n* (%)	51 (21.07)	31 (19.75)	20 (23.53)	0.491
Stroke, *n* (%)	22 (9.09)	12 (7.64)	10 (11.76)	0.287
White blood cell count, 10^9^/L	6.45 (5.10, 7.70)	6.50 (5.20, 7.60)	6.40 (5.10, 7.90)	0.649
Hemoglobin, g/L	124.64 ± 19.47	127.05 ± 17.89	120.19 ± 21.51	0.009
Albumin, g/L	39.31 ± 4.57	40.00 ± 4.03	38.03 ± 5.22	0.003
Blood urea nitrogen, mmol/L	6.00 (4.82, 7.40)	5.80 (4.70, 7.10)	6.70 (5.30, 7.80)	0.009
eGFR, mL/min	87.05 (75.56, 93.21)	89.00 (78.00, 94.00)	83.79 (66.20, 92.00)	0.006
Creatinine, µmol/L	65.00 (56.00, 75.75)	63.00 (55.00, 73.00)	67.00 (59.00, 86.00)	0.061
Glucose, mmol/L	5.31 (4.69, 6.19)	5.31 (4.71, 6.04)	5.33 (4.56, 6.33)	0.965
Potassium, mmol/L	3.93 (3.70, 4.29)	3.93 (3.71, 4.32)	3.91 (3.66, 4.23)	0.350
Sodium, mmol/L	141.5 (140.0, 143.0)	142.0 (140.0, 143.0)	141.0 (140.0, 143.0)	0.289
Neutrophil‐to‐lymphocyte ratio	2.90 (1.88, 4.78)	2.50 (1.67, 4.30)	3.63 (2.27, 5.67)	0.002
Anesthesia and surgical factors				
Fasting times for solids, h	15 (14, 18)	15 (13, 18)	16 (14, 18)	0.029
Fasting times for fluids, h	5.00 (3.00, 8.50)	5.00 (3.00, 9.00)	5.00 (3.00, 8.00)	0.716
Temperature < 36, °C	50 (20.66)	30 (19.11)	20 (23.53)	0.417
Hypotension, *n* (%)	116 (47.93)	71 (45.22)	45 (52.94)	0.251
Blood transfusion, *n* (%)	34 (14.05)	17 (10.83)	17 (20.00)	0.050
Duration of surgery, min	105 (80, 135)	105 (75, 130)	110 (80, 140)	0.209
Total fluid infusion, mL	1000 (600, 1100)	1000 (600, 1000)	1000 (600, 1400)	0.012
Blood loss, mL	30 (10, 100)	20 (10, 50)	50 (20, 100)	0.002
Remifentanil, mg	1.00 (0.50, 1.00)	1.00 (0.50, 1.00)	1.00 (0.30, 1.00)	0.878
Sufentanil, µg	30 (25, 30)	30 (25, 30)	30 (20, 30)	0.703
Use of glucocorticoids, *n* (%)	200 (82.64)	133 (84.71)	67 (78.82)	0.248
Use of dexmedetomidine, *n* (%)	154 (63.64)	96 (61.15)	58 (68.24)	0.274
Postoperative factors				
Presence of urinary catheters, *n* (%)	49 (20.25)	19 (12.10)	30 (35.29)	<0.001
NRS score	1 (0, 2)	1 (0, 2)	1.00 (1, 2)	0.004

Abbreviations: ASA, American Society of Anesthesiologists; COPD, chronic obstructive pulmonary disease; ED, emergence delirium; eGFR, estimated glomerular filtration rate; NRS, numerical rating scale.

### 2.6 Statistical Analysis and Sample Size

#### 2.6.1 Sample Size

This study employed multivariate logistic regression analysis for data processing. The sample size was determined according to the events‐per‐variable (EPV) principle, with reference to the simulation study by Peduzzi et al. (1996). Their research indicated that at least 10 EPVs are required to ensure the validity of the model. On the basis of this, this study anticipated that there would be seven independent variables ultimately included in the regression model. Therefore, the number of positive cases of ED should reach 7 × 10 = 70. According to another study, the incidence of ED after general anesthesia in elderly patients is approximately 37% (Zhang et al. 2020). Consequently, the minimum sample size was calculated as 70 ÷ 37% ≈ 189 cases. Considering a 10% sample attrition rate, the final determined sample size was at least 189 + 189 × 10% ≈ 208 cases.

#### 2.6.2 Statistical Analysis

For all continuous measures, we first applied the Shapiro–Wilk normality testing. Parameters demonstrating normal distribution were characterized by mean ± standard deviation and analyzed with Student's *t*‐test, whereas skewed distributions were described using median (interquartile range) and evaluated through Mann–Whitney *U*‐tests. Categorical data were reported as numbers and percentages, with intergroup comparisons conducted using either Fisher's exact or *χ* ^2^ tests according to data characteristics. Statistical significance was defined as a two‐tailed *p* value below 0.05.

Multicollinearity was examined through tolerance (Tol) values and variance inflation factors (VIF). Following conventional thresholds, collinearity was deemed acceptable when Tol exceeded 0.1 or VIF remained below 10. Variables attaining statistical significance (*p* < 0.05) during initial univariate screening were entered into a subsequent backward stepwise multivariate logistic regression. Association strengths were characterized by odds ratios (ORs), their 95% confidence intervals (CIs), and corresponding *p* values. Model performance was assessed by evaluating both discrimination and calibration. Discrimination, the ability of the model to differentiate between patients with and without ED, was quantified using the area under the curve (AUC). Calibration, the agreement between predicted probabilities and observed outcomes, was assessed using the Hosmer–Lemeshow goodness‐of‐fit test and a calibration plot.

Subgroup analyses were conducted to assess whether age (<80 years vs. ≥80 years), sex, hypertension, hyperlipidemia, and presence of urinary catheters influenced the association between dexmedetomidine administration and ED. A two‐sided *p* value of <0.05 was defined as indicating statistical significance. Statistical analyses were conducted with IBM SPSS Statistics (version 29.0) and R (version 4.4.3).

## 3 Results

### 3.1 Patient Characteristics

**FIGURE 1:**
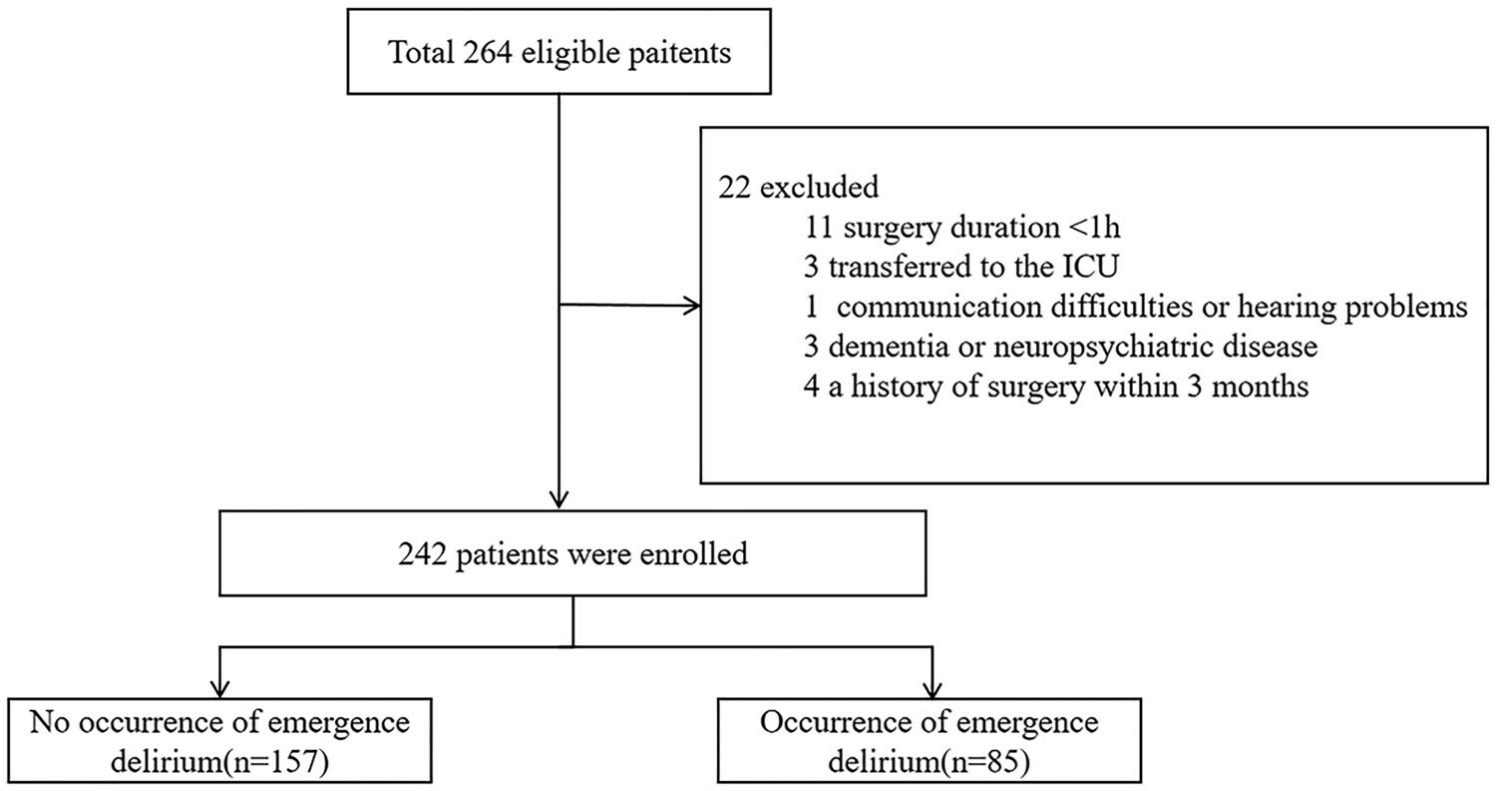
Flow chart of the study population.

In this study, 264 individuals undergoing orthopedic surgery were recruited for participation. After screening against the selection criteria, 22 patients were excluded, with 242 cases ultimately retained for analysis (Figure 1). The final recruitment figures satisfied the pre‐calculated sample size requirements for robust binary logistic regression modeling.

The observed prevalence of ED was 35.1%, as detailed in Table 1. Regarding preoperative factors, patients experiencing ED tended to be older, had a higher proportion of ASA class III, a higher prevalence of hypertension and hyperlipidemia, and higher NLR and blood urea nitrogen levels. However, their estimated glomerular filtration rate (eGFR), hemoglobin, and albumin levels were lower (all *p* < 0.05). Among anesthesia and surgical factors, the ED group had longer fasting times for solids, a greater total fluid infusion volume, and a larger blood loss (all *p* < 0.05). In terms of postoperative factors, the ED group exhibited statistically greater proportions of patients with indwelling urinary catheters and higher NRS scores relative to the non‐ED group (all *p* < 0.05). Notably, no statistically significant differences were observed between the ED and non‐ED groups in the use of key anesthetic agents, including dexmedetomidine, remifentanil, and sufentanil (all *p* > 0.05).

### 3.2 Multicollinearity Diagnostics for Candidate Variables

As demonstrated in Table S1, the Tol of each variable was within a reasonable range (>0.1), and all VIFs were far less than 10. These findings indicated that there was no significant multicollinearity among these variables.

### 3.3 Risk Factors Associated With ED

**FIGURE 2:**
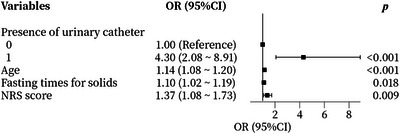
Risk factors for emergence delirium using meta‐analysis plot. Presence of urinary catheter (OR: 4.30; 95%CI: 2.08–8.91), age (OR: 1.14; 95%CI: 1.08–1.20), fasting times for solids (OR: 1.09; 95%CI: 1.02–1.17), NRS score (OR: 1.33; 95%CI: 1.08–1.64) were the independent risk factors. CI, confidence interval; NRS, numerical rating scale; OR, odds ratio.

We performed univariate logistic regression analyses to evaluate the associations of each variable, each candidate predictive variable, with ED. The results revealed that the following variables had a *p* value < 0.05: hypertension, hyperlipidemia, ASA class, presence of urinary catheters, age, hemoglobin, blood urea nitrogen, eGFR, albumin, NLR, blood loss, total fluid infusion, fasting times for solids, and NRS score (Table 2). Multivariate logistic regression incorporating these 14 variables identified four independent risk factors of ED: presence of urinary catheters (OR 4.30, 95% CI 2.08–8.91), age (OR 1.14, 95% CI 1.08–1.20), NRS score (OR 1.37, 95% CI 1.08–1.73), and fasting times for solids (OR 1.10, 95% CI 1.02–1.19), as visualized in Figure 2.

**TABLE 2:** Univariate logistic regression analysis for emergence delirium.

Variables	Odds ratio (95% CI)	*p* value
Hypertension, *n* (%)	1.91 (1.10–3.31)	0.021
Hyperlipidemia, *n* (%)	0.50 (0.28–0.90)	0.022
ASA class, *n* (%)		
I	Reference	
II	2.28 (0.83–6.29)	0.111
III	11.17 (3.02–41.31)	<0.001
Presence of urinary catheters, *n* (%)	3.96 (2.06–7.62)	<0.001
Age, year	1.13 (1.08–1.19)	<0.001
Hemoglobin, g/L	0.98 (0.97–0.99)	0.010
Blood urea nitrogen, mmol/L	1.20 (1.06–1.36)	0.004
eGFR, mL/min	0.98 (0.96–0.99)	0.002
Albumin, g/L	0.91 (0.85–0.96)	0.002
Neutrophil‐to‐lymphocyte ratio	1.09 (1.01–1.19)	0.030
Blood loss, mL	1.01 (1.01–1.01)	0.008
Total fluid infusion, mL	1.01 (1.01–1.01)	0.006
Fasting times for solids, h	1.09 (1.02–1.17)	0.016
NRS score	1.33 (1.08–1.64)	0.008

*Note*: Only the variables with *p* < 0.5 in the univariate logistic regression analysis are listed in the table.

Abbreviations: ASA, American Society of Anesthesiologists; CI, confidence interval; eGFR, estimated glomerular filtration rate; NRS, numerical rating scale.

### 3.4 Performance of the Model

**FIGURE 3:**
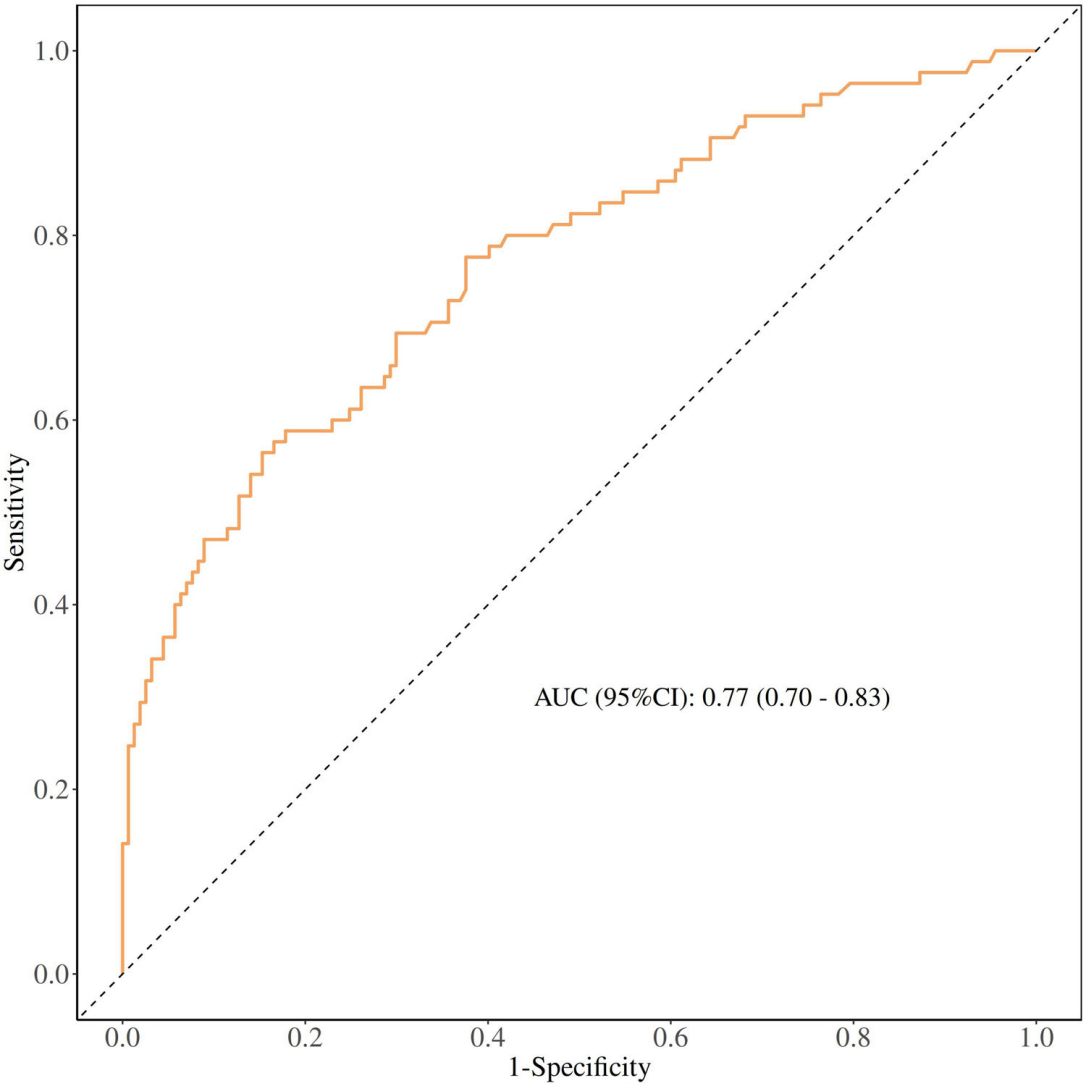
Receiver operating curve for the model of emergence delirium. AUC, area under the curve.

**FIGURE 4:**
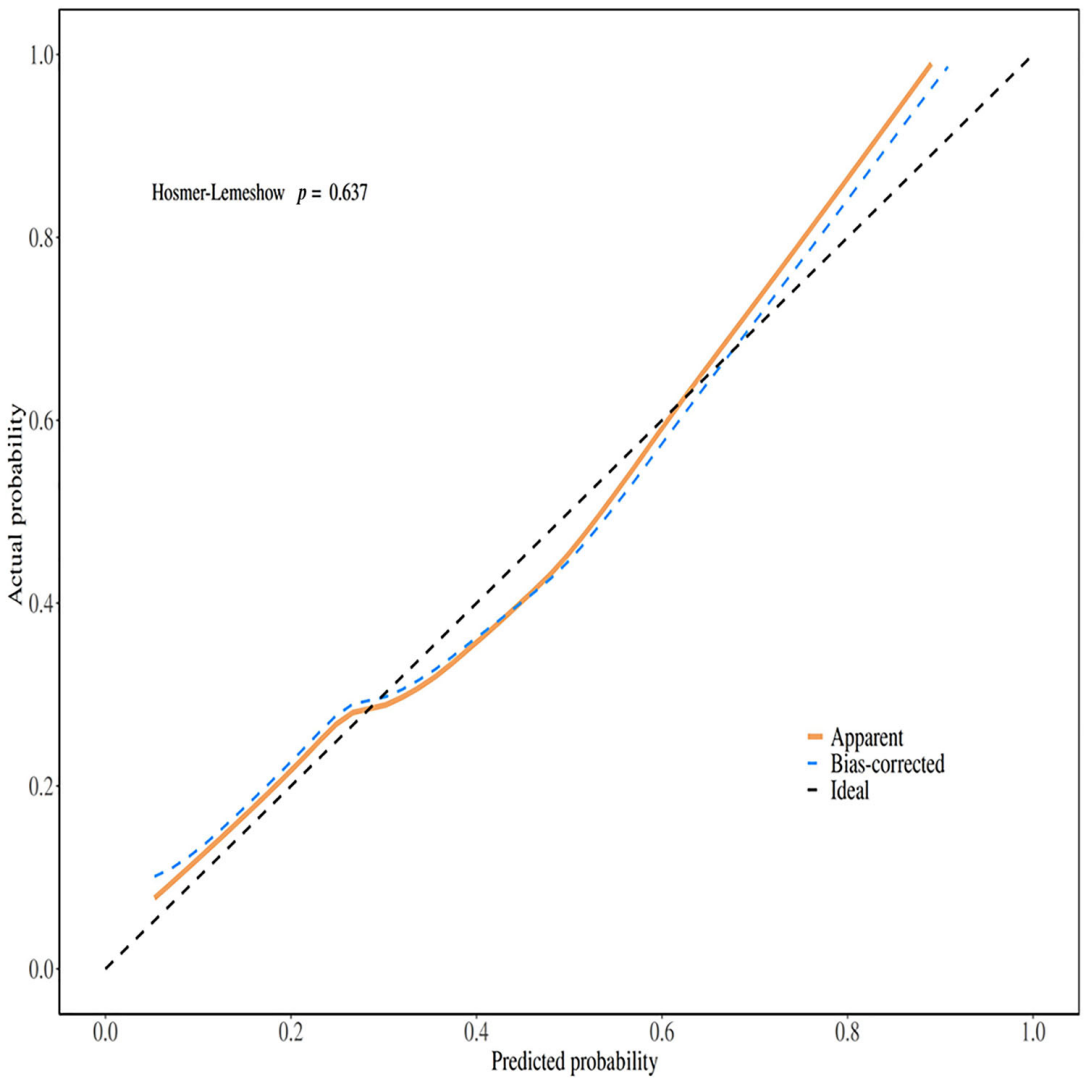
Calibration plot for the model of emergence delirium.

As shown in Figure 3, the model showed acceptable discriminatory power with an AUC of 0.77 (95% CI: 0.70–0.83). Importantly, the Hosmer–Lemeshow goodness‐of‐fit test yielded a chi‐square value of 6.09 with 8 degrees of freedom (*p* = 0.637), indicating no significant deviation between the predicted and observed probabilities. This excellent calibration was further visually confirmed by the calibration plot, where the prediction curve closely followed the ideal line (Figure 4).

### 3.5 Subgroup Analysis

**FIGURE 5:**
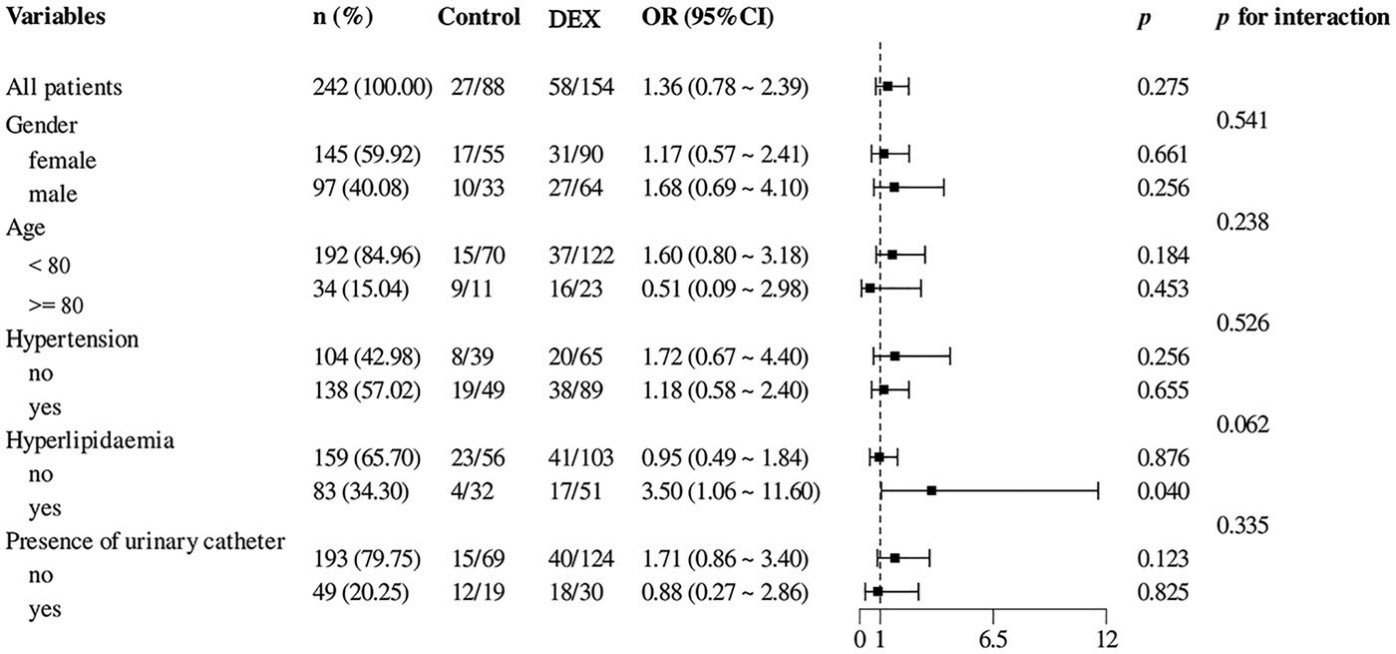
Subgroup analysis of the association between intraoperative dexmedetomidine and emergence delirium. CI, confidence interval; EDX, dexmedetomidine; OR, odds ratio.

We performed subgroup analyses to examine the association between dexmedetomidine administration and ED. As shown in Figure 5, there was no statistically significant evidence that the association between dexmedetomidine and ED was modified by age, sex, hypertension, hyperlipidemia, or presence of urinary catheters (all *p* values for interaction >0.05).

## 4 Discussion

Our prospective observational study indicates that ED is a common postoperative complication among elderly patients undergoing orthopedic surgery under general anesthesia, with an incidence rate of 35.1%. This finding aligns with existing epidemiological reports on ED (Zhang et al. 2020; Huang et al. 2020). However, most current studies mainly focus on delirium that occurs after being transferred out of the PACU (An et al. 2023; Lai et al. 2023). To the best of our understanding, this research pioneers the identification of independent risk factors of ED in aged orthopedic cohorts. The final multivariate logistic regression approach demonstrated four statistically significant independent factors for ED: advanced age, presence of urinary catheters, prolonged fasting times for solids, and a higher NRS score. Furthermore, the model demonstrated acceptable discrimination (AUC = 0.77) and, more notably, excellent calibration (Hosmer–Lemeshow test, *p* = 0.637), indicating that its predictions are reliable. Given that delirium almost invariably has a multifactorial etiology, multicomponent interventions targeting the identified risk factors are likely to hold greater promise than single interventions (Devlin et al. 2018). These findings therefore provide a crucial rationale for the early identification and management of high‐risk patients in clinical practice.

This study found a significant correlation between the presence of urinary catheters and ED (OR = 4.30), which is highly consistent with previous research. As evidenced in He et al.’s meta‐analysis (He et al. 2024), indwelling urinary catheters showed substantial association with hyperactive ED (pooled OR 3.93; 95% CI 2.27–6.79; *I* ^2^ 69%). The mechanism may be related to the urinary discomfort caused by catheter stimulation, which leads to patient restlessness. Regarding this issue, studies have shown that preoperative pharmacological interventions, such as oral tolterodine administration, can effectively alleviate catheter‐related bladder discomfort (Hur et al. 2019; Agarwal et al. 2005). Therefore, for patients who require indwelling urinary catheterization, it is recommended to implement targeted preoperative interventions. It is particularly worth noting that elderly fracture patients often need a longer indwelling catheter time due to limited mobility, which may further increase the risk of delirium. This finding suggests that clinicians should rigorously assess the indications for indwelling urinary catheterization, and for patients who do require catheterization, early removal should be considered.

The prolonged preoperative solid food fasting duration is another important finding of this study. Although ASA guidelines stipulate 2‐h clear fluid and 6‐h solid food fasting periods before surgery (Practice Guidelines for Preoperative Fasting 2017), patients in clinical practice often experience extended fasting due to factors such as surgical delays. These results align with the observational study by Xara et al. (2013), who identified prolonged fasting as a modifiable determinant of ED. Research has indicated that energy metabolism disorders play a crucial role in the pathogenesis of delirium (Wilson et al. 2020). For elderly fracture patients, the trauma‐induced stress already increases metabolic demands, and the traditional fasting protocol may further exacerbate this metabolic imbalance. Therefore, we propose optimizing fasting protocols and durations to mitigate this issue.

It is widely recognized that advanced age increases the risk of delirium (Mendez‐Martinez et al. 2021; Wang et al. 2015), and our study further demonstrates a 14% augmented risk of ED development with each additional year. We believe that aging enhances delirium susceptibility through multiple interconnected mechanisms: Older patients exhibit heightened sensitivity to external stimuli due to cerebral cortical dysfunction and age‐related neurodegenerative changes in the nervous system, whereas concurrent multimorbidity and immunosenescence reduce their resilience to perioperative stressors. Additionally, reduced vascular compliance may impair cerebral perfusion, whereas transient operative hypotension may induce brain tissue hypoxia during the operation. These together constitute the pathophysiological basis for the occurrence of delirium (Hu and Yang 2023). Age‐related pharmacokinetic alterations further compound this risk: Diminished hepatic metabolism and renal excretion in elderly patients prolong anesthetic drug retention in the central nervous system by reducing clearance rates (Klotz 2009). These findings underscore the imperative of implementing precision anesthesia management in geriatric patients undergoing general anesthesia, including real‐time monitoring of anesthetic depth and individualized pharmacotherapy protocols.

Our research indicates that there is a positive correlation between postoperative pain (NRS score) and ED. This finding aligns with a recent multicenter observational study in elderly patients undergoing elective surgery, which identified postoperative pain as an independent risk factor for ED (adjusted odds ratio [AOR] 3.10, 95% CI 2.07–9.84) (Tesfaye et al. 2022). Pain, as an unpleasant emotional experience, induces complex neurobehavioral effects such as agitation (Menser and Smith 2020). Post‐orthopedic surgery pain is highly prevalent, with several studies showing suboptimal pain management in approximately half of patients postoperatively (Chan et al. 2013; Dimitriou et al. 2018). As evidenced by the study of Vaurio et al. (2006), both pain and pain management strategies constitute significant variables influencing delirium incidence among elderly surgical individuals, and pain control after surgery can reduce the risk of delirium. These findings strongly advocate for the clinical implementation of multimodal analgesia protocols to optimize postoperative pain management, particularly in orthopedic populations at heightened risk for both pain and delirium.

In contrast to the findings of several previous studies conducted in surgical settings (Li et al. 2023, 2024; Aldecoa et al. 2024), our analysis did not demonstrate a significant protective association between intraoperative dexmedetomidine use and ED in elderly orthopedic patients. This nonsignificant result remained consistent across all subgroups, as evidenced by nonsignificant interaction terms (all *p* values for interaction >0.05). This discrepancy may stem from multiple factors. First, the dosing regimen of dexmedetomidine in our study—initiated without a loading dose and maintained as a continuous infusion at 0.2–0.5 µg/kg/h for intraoperative sedation rather than delirium prophylaxis—may have been insufficient to produce a clinically meaningful effect. Second, the relatively limited sample size in some subgroups may have resulted in inadequate statistical power to detect significant interaction effects or modest treatment benefits. Therefore, future studies should aim to expand sample sizes and adjust the dexmedetomidine administration protocol.

This study has several limitations. First, its single‐center design may limit the generalizability of the findings, warranting future validation in multicenter studies. Second, the potential for misclassification may have been introduced due to the absence of a formal psychiatric evaluation using the Diagnostic and Statistical Manual of Mental Disorders criteria as the gold standard for delirium confirmation. Third, this study did not classify ED subtypes; future research should use tools like the Richmond Agitation–Sedation Scale after a positive Nu‐DESC screen to investigate subtype‐specific risks and interventions. Finally, the lack of preoperative cognitive assessment (e.g., using the Mini‐Mental State Examination or Montreal Cognitive Assessment) is a notable limitation. As preoperative cognitive impairment is a well‐established risk factor for postoperative delirium, the absence of these data may have introduced unmeasured confounding. For instance, we cannot rule out the possibility that patients with undiagnosed cognitive decline at baseline were more susceptible to delirium. Future studies should therefore incorporate baseline cognitive evaluation to better control for this potential confounder.

## 5 Conclusion

This prospective study demonstrates that ED is prevalent among elderly patients undergoing orthopedic procedures with general anesthesia. The presence of urinary catheters, prolonged preoperative solid food fasting, advanced age, and postoperative pain were determined to be independent risk factors of ED. Multimodal clinical strategies, including targeted preoperative interventions, optimized fasting protocols, precise anesthesia management, and comprehensive pain control, may mitigate the incidence of ED.

## Author Contributions

**Yufan Lu**: writing – review and editing, writing – original draft, supervision, methodology, data curation, conceptualization. Lin **Wang**: writing – original draft, formal analysis, methodology, investigation, data curation. **Ying Wang**: methodology, formal analysis, data curation. **Beiyan Ruan**: software, data curation. **Guangtao Lu**: methodology, data curation. **Xuezheng Lin**: writing – original draft, software, methodology, investigation, data curation.

This study was supported by the grants for the Medical Science and Technology Project of Zhejiang Province (Grant 2024KY1814).

## Conflicts of Interest

The authors declare no conflicts of interest.

## Supporting information




**Supplementary material**: brb371047‐sup‐0001‐SuppMat.docx

## Data Availability

The raw data supporting the findings of this study are available from the corresponding author upon reasonable request.
