# Supplementary material for: Risk Factors for Emergence Delirium in Elderly Orthopedic Patients After General Anesthesia
Source: Brain Behav. 2025 Nov 10;15(11):e71047. doi: 10.1002/brb3.71047 (PMC12602259; doi:10.1002/brb3.71047)
Supplement: Supplementary file 1 — Supplementary material: brb371047‐sup‐0001‐SuppMat.docx [file BRB3-15-e71047-s001.docx]

**Supplementary Table 1** | Collinearity analysis of all related variables

| Collinear statistics | | |
| --- | --- | --- |
| Variables | Tol | VIF |
| Gender | 0.506 | 1.977 |
| Age | 0.522 | 1.914 |
| Body mass index | 0.709 | 1.411 |
| Current smoker | 0.691 | 1.448 |
| Current drinker | 0.689 | 1.451 |
| Education | 0.772 | 1.295 |
| Coronary artery disease | 0.803 | 1.246 |
| Arrhythmia | 0.868 | 1.153 |
| Hypertension | 0.611 | 1.637 |
| COPD | 0.769 | 1.301 |
| Stroke | 0.853 | 1.172 |
| Diabetes mellitus | 0.676 | 1.480 |
| Hyperlipidemia | 0.783 | 1.277 |
| Hemoglobin | 0.421 | 2.374 |
| White blood cell count | 0.761 | 1.314 |
| Potassium | 0.617 | 1.620 |
| Sodium | 0.772 | 1.295 |
| Glucose | 0.665 | 1.504 |
| Blood urea nitrogen | 0.450 | 2.224 |
| Creatinine | 0.467 | 2.143 |
| eGFR | 0.301 | 3.325 |
| Albumin | 0.478 | 2.091 |
| Neutrophil-to-lymphocyte ratio | 0.590 | 1.696 |
| ASA class | 0.578 | 1.729 |
| Duration of surgery | 0.547 | 1.827 |
| Blood loss | 0.307 | 3.254 |
| Total fuid infusion | 0.274 | 3.651 |
| Blood transfusion | 0.424 | 2.359 |
| Hypotension | 0.800 | 1.251 |
| Temperature < 36, ℃ | 0.806 | 1.240 |
| Sufentanil | 0.803 | 1.246 |
| Remifentanil | 0.818 | 1.223 |
| Use of dexmedetomidine | 0.806 | 1.240 |
| Use of glucocorticoid | 0.836 | 1.196 |
| Fasting times for fluids | 0.804 | 1.244 |
| Fasting times for solids | 0.823 | 1.215 |
| Presence of urinary catheter | 0.684 | 1.463 |
| NRS score | 0.798 | 1.253 |

Note: COPD, chronic obstructive pulmonary disease; eGFR, estimated glomerular filtration rate; ASA, American Society of Anesthesiologists; NRS, numerical rating scale; Tol:Tolerance; VIF:Variance inflation factor.
